# Comment on “Heat transfer fluids: amino acid anion ionic liquid based IoNanofluids with remarkable thermal conductivity and low viscosity” by A. Chandra, Y. S. Sistla, M. A. Ahmed, D. V. S. Vasireddy, N. Jaglan, N. K. Das, T. Banerjee and V. S. Sistlad, *RSC Adv.* 2025, **15**, 23146

**DOI:** 10.1039/d5ra09366j

**Published:** 2026-07-08

**Authors:** Marzena Dzida, Anna Kolanowska, Krzysztof Cwynar, Katarzyna Kaczmarek

**Affiliations:** a University of Silesia in Katowice, Institute of Chemistry Szkolna 9 40-006 Katowice Poland marzena.dzida@us.edu.pl

## Abstract

Recently, Chandra *et al.* reported results of thermal conductivity and viscosity of ionanofluids (INFs) composed of 0.05 wt% multi-walled carbon nanotubes (MWCNTs) and 1-butyl-3-methylimidazolium glycinate, 1-butyl-3-methylimidazolium arginate, 1-ethyl-3-methylimidazolium glycinate or 1-ethyl-3-methylimidazolium arginate. However, the complete characterization of the ionic liquids, including purity analysis, full characterization of MWCNTs, and a comprehensive description of the used experimental methods was not provided. The inconsistent and unreliable results were not critically analyzed and compared with the available literature data. Finally, the results were presented in a biased manner by comparing them with the properties of INFs based on 1-butyl-3-methylimidazolium tetrafluoroborate. In this comment article, the analysis of the results reported by Chandra *et al.* is presented. Additionally, the approach to a comprehensive conduct of research on INFs is demonstrated.

## Abstract 2

The analysis of the results reported by Chandra *et al.*, *RSC Adv.*, 2025, **15**, 23146–23164 is presented. The approach to a comprehensive conduct of research on ionanofluids is demonstrated.

## Graphical abstract

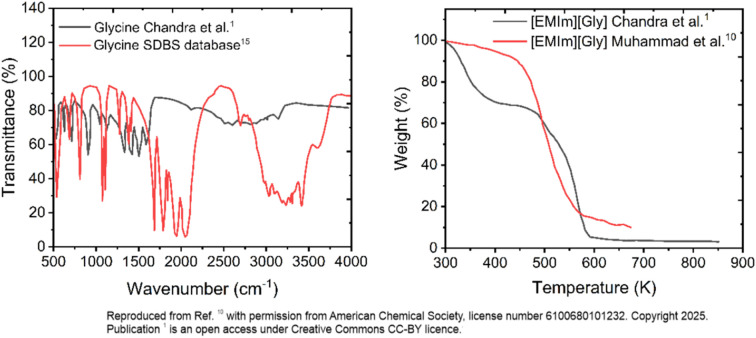

## 1. Introduction

Ionanofluids (INFs) are stable systems composed of nanoparticles dispersed in ionic liquids (ILs). Ionic liquids are composed of organic cations and inorganic or organic anions, with a somewhat arbitrary melting point below 373.15 K. Moreover, the “[TF_2_N]^−^” and “[(CF_3_SO_2_)_2_N]^−^”^1^ denote the same bis(trifluoromethylsulfonyl)imide anion, which is not the “bis(trifluoromethylsulfonyl)” anion.^1^ Mostly, ILs are treated both as a continuous phase and as a surfactant in INFs. However, Chandra *et al.*^1^ studied the effect of loading 0.05 wt% of different surfactants on the density, uniformity of dispersion, *i.e.* size of agglomerates, and sedimentation stability of INFs composed of 0.05 wt% MWCNTs and 1-butyl-3-methylimidazolium tetrafluoroborate ([BMIm][BF_4_]). The studied surfactants were cetyltrimethylammonium bromide (CTAB), lecithin/l-α-phosphatidylcholine (from soybean), sodium dodecyl sulphate (SDS), and polyoxyethylene (20) sorbitan monooleate (Tween 80). CTAB was chosen as the most suitable one. Subsequently, the effect of MWCNTs concentration and CTAB on the viscosity, specific isobaric heat capacity, thermal conductivity, and thermal stability of [BMIm][BF_4_]-based INFs were examined. Finally, all above physicochemical properties were also studied for INFs composed of 0.05 wt% MWCNTs and 1-butyl-3-methylimidazolium glycinate ([BMIm][Gly]), 1-butyl-3-methylimidazolium arginate ([BMIm][Arg]), 1-ethyl-3-methylimidazolium glycinate ([EMIm][Gly]) or 1-ethyl-3-methylimidazolium arginate ([EMIm][Arg]), with or without CTAB.

We have decided to write this comment, because R. P. Feynman wrote: “…*if you're doing an experiment, you should report everything that you think might make it invalid—not only what you think is right about it: other causes that could possibly explain your results; and things you thought of that you've eliminated by some other experiment, and how they worked—to make sure the other fellow can tell they have been eliminated. Details that could throw doubt on your interpretation must be given, if you know them. You must do the best you can—if you know anything at all wrong, or possibly wrong—to explain it*”.^2^ In comment, we omitted the discussion on the addition of surfactants due to the lack of available literature data.

## 2. Literature review

An important part of the research is the literature review on the subject of the study. Chandra *et al.*^1^ studied INFs composed of (0.025–0.1 wt%) MWCNTs (diameter 37–53 nm, length was not specified, purity 95 wt%) and [BMIm][BF_4_]. However, the works concerning the same type of INFs were not cited. Minea and Cherecheş^3^ reported the thermal conductivity of the analogical INFs composed of 0.025–0.1 wt% MWCNTs (diameter 50–90 nm, length was not specified, purity 95 wt%) and [BMIm][BF_4_] in the temperature range of 293.15–333.15 K. Cherecheş *et al.*^4^ studied viscosity variation with shear rate at 298.15 K, and specific isobaric heat capacity at 293.15 K of the similar system of (0.025–0.01 wt%) MWCNTs (diameter 50–90 nm, length was not specified, purity 95 wt%) and [BMIm][BF_4_]. The work of Nieto de Castro *et al.*,^5^ who reported thermal conductivity of INFs composed of 1 wt% MWCNTs (diameter 13–16 nm, length 1–10 µm, purity >99 wt%) and [BMIm][BF_4_] at room temperature was also not cited. The [BMIm][BF_4_] is one of the most popular IL and complete set of thermophysical properties can be found in IL Thermo database.^6^ Thus, the properties of [BMIm][BF_4_] could be compared with the literature data. Moreover, the properties of [EMIm][Gly] are relatively well described in the literature. Bai *et al.*^7^ reported density in the temperature range of 288.15–343.15 K, viscosity in the temperature range of 288.15–343.15 K, water content, Fourier transform infrared spectroscopy (FT-IR) spectrum and proton nuclear magnetic resonance (^1^H-NMR) spectrum of [EMIm][Gly]. Chen *et al.*^8^ reported viscosity of [EMIm][Gly] in the temperature range from 298.15 to 353.15 K. Gouveia *et al.*^9^ reported density, viscosity, and refractive index in the temperature range of 293.15–353.15 K, onset and decomposition temperatures, water content in [EMIm][Gly] as well as ^1^H-NMR spectrum of [EMIm][Gly] in DMSO-*d*_6_ and carbon-13 nuclear magnetic resonance (^13^C-NMR) spectrum of [EMIm][Gly] in DMSO-*d*_6_. Muhammad *et al.*^10^ reported density, viscosity, refractive index, and isobaric thermal expansion coefficient in the temperature range of 293.15–353.15 K, onset and decomposition temperatures, and water content in [EMIm][Gly]. All of the above-mentioned papers concerning the characterization and thermophysical properties of [EMIm][Gly] can also be found in the IL Thermo database.^6^

## 3. Experimental methods

### 3.1. Characterization of MWCNTs

Chandra *et al.*^1^ reported supplier (Sigma-Aldrich) purity and diameter of MWCNTs. The reported diameter of 37–53 nm was obtained from scanning electron microscopy, but without any information about the number of carbon nanotubes (CNTs) used for statistical analysis. The length of the studied MWCNTs was not reported. Based on the above information two types of MWCNTs, offered by Sigma-Aldrich, can be formally selected (Table 1). These MWCNTs differ in both length and diameter range, which in turn affects the aspect ratio (the ratio of their length to their diameter). The aspect ratio has a substantial influence on the properties of CNTs, including thermal conductivity, which is the primary focus of the Chandra's *et al.*^1^ work. The greater aspect ratio leads to higher thermal conductivity of the INFs at a constant MWCNTs content.^11,12^

**Table 1:** Characteristics of MWCNTs available on the Sigma-Aldrich website (date of access 30.09.2025) consistent with the characteristics of CNTs described by Chandra *et al.*^1^

Number	Diameter (nm)	Length (µm)	Purity (wt%)	Functionalities
901019	50–90	5–9	>95	Pristine
901002	10–40	0.5–1.5	>95	Pristine

**Fig. 1:**
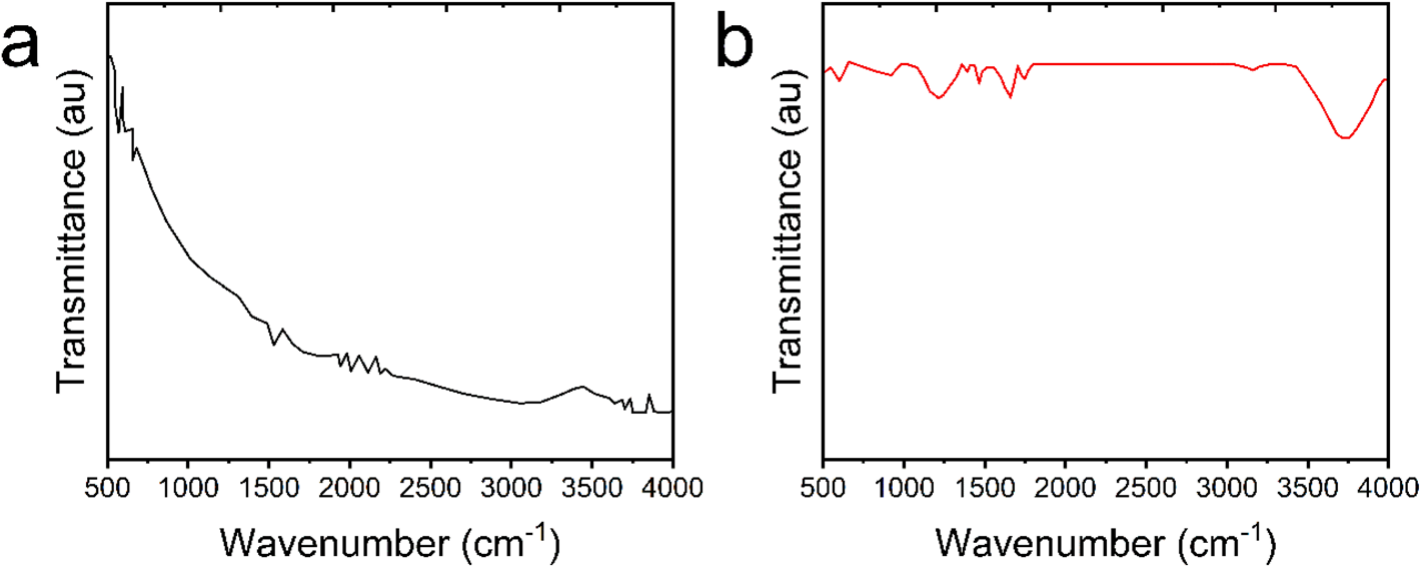
Comparison of FT-IR spectra of (a) MWCNTs (purity >95 wt%, diameter 37–53 nm, length not specified) reported by Chandra *et al.*;^1^ and (b) MWCNTs (purity >98 wt%, diameter 9.5 nm, length 1.5 µm) reported by González-Domínguez *et al.*^13^ Fig. 1b reproduced from ref. 13 with permission from Royal Society of Chemistry, license number 1656897. Copyright 2025. Publication^1^ is an open access under Creative Commons CC-BY licence.

In the case of CNTs, for characterization of the research material the following procedures are essential: (i) diameter measurement of the CNTs using scanning or transmission electron microscopy. To ensure scientific relevance, statistical analysis should be performed on a minimum of one hundred individual nanotubes; (ii) length measurement of the CNTs, *via* scanning or transmission electron microscopy, based on a sufficiently large number of objects to ensure statistical significance; (iii) thermogravimetric analysis (TGA), which should be conducted in both inert gas and air atmospheres. Analysis under nitrogen or argon provides insight into the thermal stability and quality of the material. The CNTs may be contaminated with other carbon forms, such as amorphous or glassy carbon. Performing the analysis in air enables the assessment of residual metallic catalyst content used during the synthesis of the nanomaterial; (iv) Raman spectroscopy, which allows the evaluation of the nanomaterial's quality through the analysis of the graphitic mode (related to the presence of sp^2^-hybridized carbon) and the defect mode (associated with sp^3^-hybridized carbon). While, Chandra *et al.*^1^ chose FT-IR spectroscopy for the analysis of CNT samples instead, which presents a substantial technical challenge. The CNTs strongly absorb infrared radiation across the spectrum, necessitating the use of a potassium bromide (KBr) pellet for transmission-mode measurements. However, Chandra *et al.*^1^ provide no details regarding the preparation of the CNT sample for this analysis. For accurate and meaningful results, significant dilution of the sample is essential to achieve sufficient transparency, and anhydrous KBr must be used. Due to the strongly hygroscopic nature of KBr, all sample preparation should be carried out in a glove box. Failure to do so results in the pellet becoming cloudy and the FT-IR spectrum being dominated by water absorption bands, rendering it unreliable. The FT-IR spectra of MWCNTs reported by Chandra *et al.*^1^ was compared with those of reported by González-Domínguez *et al.*^13^ in Fig. 1. These FT-IR spectra were compared only qualitatively, as the transmittance axis lacked a scale for both works.^1,13^

The FT-IR spectra of MWCNTs reported by Chandra *et al.*^1^ (Fig. 1a) and González-Domínguez *et al.*^13^ (Fig. 1b) show notable differences in both spectral quality and band assignment. The FT-IR spectrum in Fig. 1a exhibits two main peaks at 3450 cm^−1^ and 1550 cm^−1^, corresponding respectively to O–H stretching (water contamination) and C=C stretching of MWCNTs, it also displays significant noise and inverted or barely visible bands, particularly in the 1200–1000 cm^−1^ fingerprint region and around 2000–2250 cm^−1^. In contrast, the spectrum in Fig. 1b, representing high-purity MWCNTs (98 wt%), exhibits well-defined and characteristic bands. The peaks at 2923 cm^−1^ and 2834 cm^−1^ correspond to Csp^3^–H stretching due to trace aliphatic contaminants, while those at 1560 cm^−1^ and 1629 cm^−1^ represent the C=C phonon modes of graphitic sheets and adsorbed moisture, respectively. The O–H stretching band appears at 3442 cm^−1^, and the signal at 1150 cm^−1^ indicates C–O stretching vibrations from surface oxygen groups. The comparison clearly shows that the spectrum presented by Chandra *et al.*^1^ lacks the expected spectral features, which are observed in the spectrum reported by González-Domínguez *et al.*^13^ Notably, FT-IR spectroscopy of pristine MWCNTs is inherently challenging because the ideal graphitic lattice possesses few infrared-active fundamental vibrations. For MWCNTs, FT-IR analysis is mainly used to identify residual impurities from synthesis, molecules adsorbed or covalently attached to the nanotube surface, and the presence of functional groups. The most prominent spectroscopic signatures of carbon nanomaterials are Raman features (D and G bands) rather than intense infrared absorptions. Accordingly, Raman spectroscopy remains one of the most powerful and informative techniques for MWCNTs characterization.^14^

### 3.2. Characterization of ionic liquids

#### 3.2.1 FT-IR spectra

**Fig. 2:**
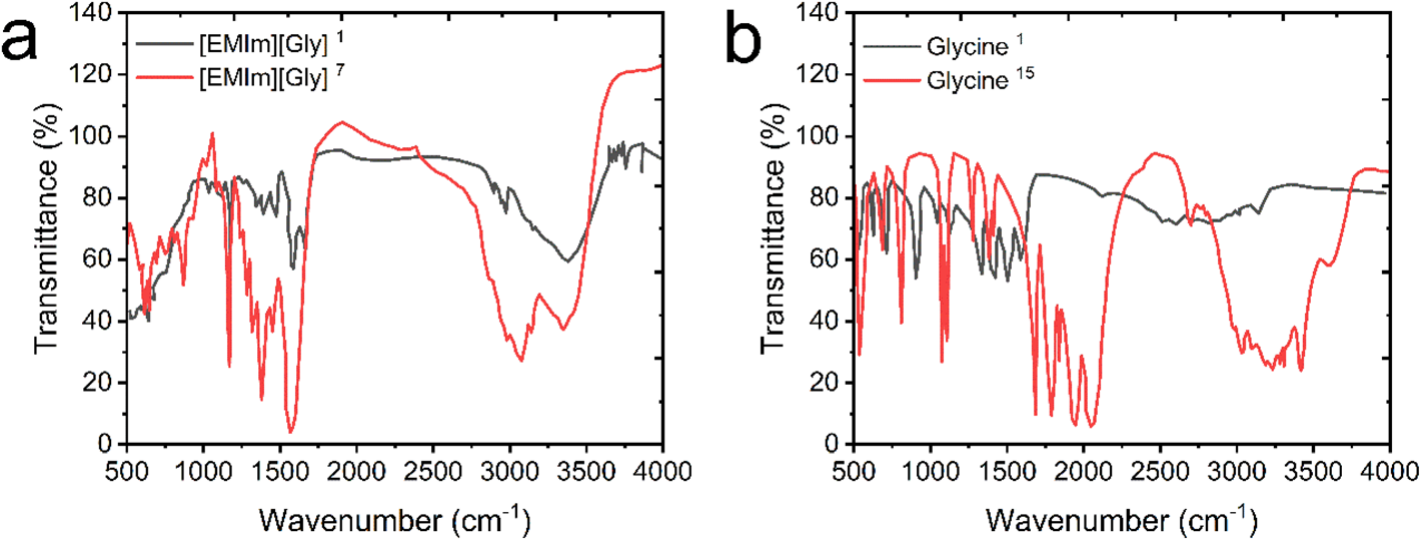
Comparison of FT-IR spectra of (a) [EMIm][Gly] reported by Chandra *et al.*^1^ (black curve) and Bai *et al.*^7^ (red curve), and of (b) glycine from spectral database for organic compounds^15^ (black curve) and reported by Chandra *et al.*^1^ (red curve). Reproduced from ref. 7 with permission from American Chemical Society, license number 6100671348331. Copyright 2025. Publication^1^ is an open access under Creative Commons CC-BY licence.

For characterization of the synthesized ILs Chandra *et al.*^1^ also chose FT-IR method. The FT-IR spectra provide information about the functional groups present in the compound. However, it is not a technique that offers definitive insights into molecular structure. It can serve such a purpose only when the fingerprint region is compared with a reference sample that has been previously characterized under identical measurement conditions. The Fig. 2a presents a comparison between two FT-IR spectra of the [EMIm][Gly], one derived from Chandra *et al.*^1^ (black curve) and from Bai *et al.*^7^ (red curve). While both spectra aim to characterize the same compound, they differ markedly in spectral quality, resolution, and reproducibility.

The spectrum of [EMIm][Gly] reported by Bai *et al.*^7^ (red line) exhibited well-defined and sharp absorption bands throughout the mid-infrared range (4000–500 cm^−1^). Distinct features corresponding to functional groups of the [EMIm][Gly] are clearly resolved. These include bands in the region around ∼3100–3000 cm^−1^, attributable to the C–H stretching of the imidazolium ring, and a broad band near ∼3400 cm^−1^ likely arising from O–H or N–H stretching vibrations. Strong, well-resolved absorptions in the 1700–1400 cm^−1^ region correspond to asymmetric and symmetric stretching of carboxylate (–COO^−^) groups, a characteristic feature of the glycinate anion. Fingerprint region features (∼1500–500 cm^−1^) are likewise discernible and consistent with known vibrational modes of the imidazolium cation and glycinate anion. In contrast, the spectrum presented by Chandra *et al.*^1^ (black line) displays several shortcomings indicative of poor spectral quality (Fig. 2a). The overall baseline is unstable and noisy, particularly in the fingerprint region. Band resolution is significantly lower, with broader, less distinct peaks. Several features that are prominent in the spectrum reported by Bai *et al.*^7^ are either severely diminished or altogether absent in the spectrum reported by Chandra *et al.*^1^ The high noise-to-signal ratio and lack of symmetry in key vibrational bands affect the reliability of the measurements. Furthermore, the transmittance scale in the spectrum is inconsistent, as the data fluctuates irregularly around certain regions (*e.g.*, 1000–1700 cm^−1^),^1^ whereas the spectrum reported by Bai *et al.*^7^ maintains a physically consistent baseline and peak intensity profile. In summary, while both spectra nominally represent the same IL, the spectrum reported by Bai *et al.*^7^ clearly demonstrates higher spectral fidelity, accurate peak assignment, and reproducibility, reflective of rigorous experimental practices. Additionally, the FT-IR spectrum obtained by Chandra *et al.*^1^ for glycine was compared with the reference spectrum available in a spectral database for organic compounds (SDBS) (Fig. 2b).^15^ The FT-IR spectra of glycine from SDBS exhibit characteristic absorption features associated with its functional groups, most notably the amino and carboxylic moieties. The key vibrational modes are clearly resolved. The strong band observed near 3400–3200 cm^−1^ corresponds to N–H stretching vibrations of the protonated amino group. In addition, the C=O stretching mode of the carboxylate appears as a prominent absorption near 1600 cm^−1^, accompanied by symmetric COO^−^ stretching around 1410 cm^−1^. The fingerprint region (below 1300 cm^−1^) is marked by a series of well-defined absorptions attributed to C–N stretching, and C–H bending vibrations, characteristic of glycine's zwitterionic form. In spectrum reported by Chandra *et al.*^1^ (black curve in Fig. 2b) the baseline appears poorly corrected, and the signal-to-noise ratio is significantly lower, obscuring weaker but diagnostically important features. The N–H stretching region is broadened and lacks the distinct separation seen in the reference spectrum. Moreover, the carboxylate stretching bands are either shifted or ill-defined. The fingerprint region is especially problematic, with diffuse and overlapping signals that do not reproduce the fine structure documented in the reference dataset. Generally, the products of the synthesis are typically confirmed using nuclear magnetic resonance (NMR) spectroscopy, including both ^13^C and ^1^H NMR.

#### 3.2.2 Thermogravimetric analysis

**Fig. 3:**
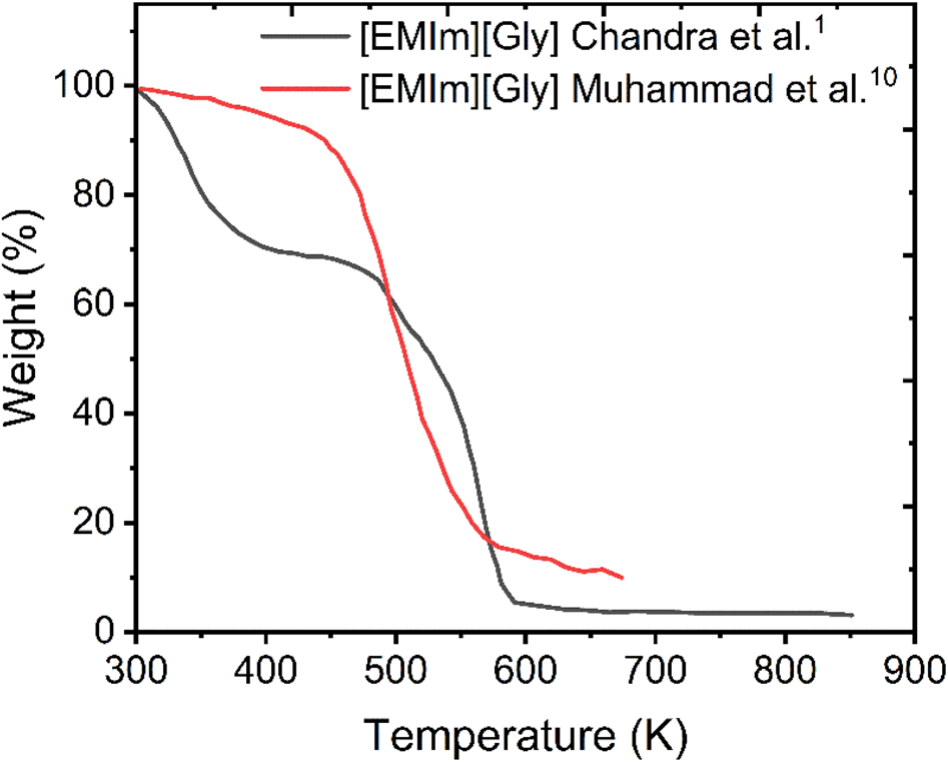
Comparison of TGA curves of [EMIm][Gly] obtained by Chandra *et al.*^1^ (black curve) and by Muhammad *et al.*^10^ (red curve). Reproduced from ref. 10 with permission from American Chemical Society, license number 6100680101232. Copyright 2025. Publication^1^ is an open access under Creative Commons CC-BY licence.

The efficiency of purification process of the ILs after synthesis can be examined by TGA. The Fig. 3 shows the comparison of TGA curves performed by Chandra *et al.*^1^ (black line) and by Muhammad *et al.*^10^ (red line).

The most significant differences between these two curves are observed in the initial mass loss below 473 K and in the onset of the main decomposition. The results reported by Chandra *et al.*^1^ (black curve in Fig. 3) show a substantial weight loss in the region below 423 K, dropping by approximately 30%. This is strongly indicative of volatile impurities, most likely water or residual solvents that were not removed during purification. In contrast, the sample described by the red curve remains more stable up to around 443–453 K, with the weight loss <5.0 wt%. In the main decomposition region, the sample described by the red curve in Fig. 3 displays a relatively sharp and consistent degradation profile, indicating a single, well-defined decomposition process, typical of a pure product. While, the sample described by the black curve in Fig. 3, exhibits a broader and less distinct degradation range, which may reflect multiple overlapping decomposition events. The synthesis took place in an aqueous environment, after which unreacted amino acids were removed *via* precipitation, and residual solvents were eliminated using a rotary evaporator. However, the hygroscopic ILs retain water, which is impossible to remove by rotary evaporation alone. The [EMIm][Gly],^10,16^ as well as several other ILs derived from amino acids, have been previously synthesized, and vacuum oven drying was deemed necessary.^10,16,17^ The presence of water in the samples is further supported by the TGA results, which show a significant mass loss below 373 K. This indicates water content – especially when compared to TGA profiles of ILs that were properly dried in a vacuum oven (see Fig. 3). Chandra *et al.*^1^ provided no information about the water content, which was a crucial in the context of evaluating the viscosity, isobaric heat capacity, thermal conductivity, and density of INFs. Thus, the statement provided by Chandra *et al.*^1^ that studied ILs derived from amino acids (AAILs, Amino Acids Ionic Liquids) were stable up to 573 K was somewhat optimistic (see Fig. 4a in Chandra *et al.*^1^).

**Fig. 4:**
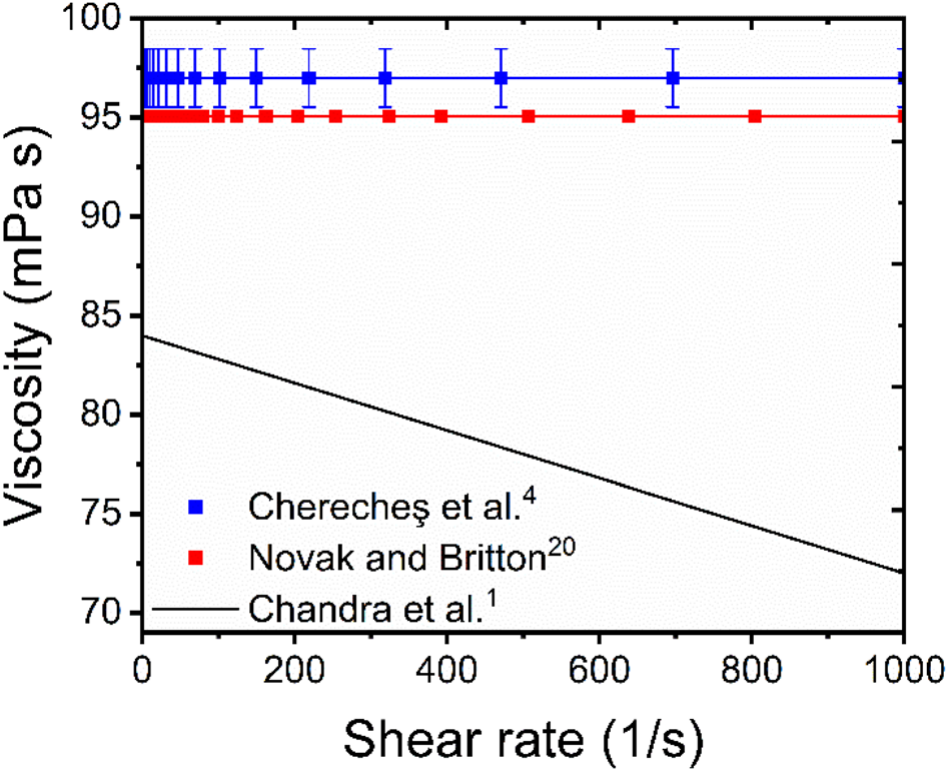
Comparison of the flow curve for [BMIm][BF_4_] at 298 K reported by Cherecheş *et al.*,^4^ Novak and Britton^20^ and Chandra *et al.*^1^

#### 3.2.3 Differential scanning calorimetry

The stability of AAILs up to 573 K was also not confirmed by differential scanning calorimetry (DSC). Chandra *et al.*^1^ found the first broad endotherm effect between 278–443 K having a peak at around 369–381 K for all four AAILs (see Fig. 4b in Chandra *et al.*^1^). This effect was interpreted as the gradual phase transition due to melting. However, the authors reported the melting temperature of all AAILs as ∼372.15 K. Chandra *et al.*^1^ found the second endotherm between 493–543 K, having a peak at 523 K, related to the decomposition of all four AAILs (see Fig. 4b in Chandra *et al.*^1^). Second event attributed, by Chandra *et al.*,^1^ to decomposition was small and narrow in comparison to the one interpreted as melting. Moreover, the literature data cited by Chandra *et al.*^1^ did not confirm such high stability up to 573 K of these IL types.

#### 3.2.4 Density

Chandra *et al.*^1^ decided to conduct density measurement in semi-quantitative way by weight of known volume of IL or INF. However, the temperature was not specified and uncertainty of the density measurements was not provided. The presented results are an average of five measurements, but statistics were not reported. Chandra *et al.*^1^ compared the obtained density with the literature data. However, Chandra *et al.*^1^ incorrectly reported value of the experimental density of [BMIm][NTf_2_], which has been conducted by Kanakubo and Harris.^18^ Temperature was not specified and the number of significant places was reduced in comparison with the original experimental data of [BMIm][NTf_2_].^18^ Based on the density values cited by Chandra *et al.*,^1^ it was difficult to determine the temperature to which these values refer (see Table 2). For [BMIm][BF_4_] the value of density cited by Chandra *et al.*^1^ has not been reported by Li *et al.*^19^ (see Table 2).

**Table 2:** Density of [BMIm][NTf_2_] and [BMIm][BF_4_]

*T* (K)	*ρ* × 10^−3^ (kg m^−3^)	Literature source
**[BMIm][NTf**_**2**_ **]**		
Not specified	1.44	Kanakubo and Harris^18^ cited by Chandra *et al.*^1^
293.15	1.44143	Kanakubo and Harris^18^
293.15	1.44142	Kanakubo and Harris^18^
298.15	1.43664	Kanakubo and Harris^18^
298.15	1.43660	Kanakubo and Harris^18^

**[BMIm][BF**_**4**_ **]**		
Not specified	1.21	Li *et al.*^19^ cited by Chandra *et al.*^1^
303.15	1.1961	Li *et al.*^19^
308.15	1.1926	Li *et al.*^19^
313.15	1.189	Li *et al.*^19^
318.15	1.1855	Li *et al.*^19^
323.15	1.182	Li *et al.*^19^
278.15–289.15^a^	1.21^b^	Calculated in this work based on the data reported by Li *et al.*^19^

a Extrapolated value.

b Extrapolated value calculated from eqn (1).^19^

#### 3.2.5 Viscosity

Chandra *et al.*^1^ mentioned only the type of apparatus used for viscosity measurements (rheometer MCR302e, Anton Paar, Austria). While, the viscosity values are strictly dependent on the measurement procedure, *i.e.* the geometry used, gap size, and sample handling during viscosity measurement. The uncertainty of the viscosity measurements was also not reported. Moreover, the raw viscosity data points have not been reported, which requires readers to digitize the data from figures using appropriate software. In the measurement procedures and equipment characterization section (2.4.3. paragraph in Chandra *et al.*^1^) Chandra *et al.*^1^ stated shear rate range as 0.1–1000 s^−1^. On the other hand, in the results section (3.3.3. paragraph in Chandra *et al.*^1^) shear rate range for [BMIm][BF_4_] and [BMIm][BF_4_]-based INFs was reported to be 1–1000 s^−1^, which was presented in Fig. 6a in Chandra *et al.*^1^ We used a shear rate range of 1–1000 s^−1^ for the digitalization of Fig. 6a in Chandra *et al.*^1^ The Fig. 4 presents the viscosity variation at different shear rates for [BMIm][BF_4_] reported by Cherecheş *et al.*,^4^ Novak and Britton,^20^ and Chandra *et al.*^1^ Cherecheş *et al.*^4^ measured viscosity of [BMIm][BF_4_] at 298.15 K as function of shear rate from 1–1000 s^−1^ with parallel plate (diameter 50 mm, gap 0.5 mm) by rheometer Physica MCR 501 (Anton Paar, Austria). Novak and Britton^20^ measured viscosity of [BMIm][BF_4_] at 298 K as function of shear rate from 0.05–1000 s^−1^ with cone-plate geometry (cone diameter 60 mm, cone angle 2°) by AR-G2 rheometer (TA Instruments).

Cherecheş *et al.*^4^ and Novak and Britton^20^ found that [BMIm][BF_4_] at 298 K is Newtonian liquid using different rheometers and different geometries. While, in Chandra *et al.*,^1^ a steady decrease in viscosity of [BMIm][BF_4_] can be observed, up to 14%, with an increasing shear rate from 1 s^−1^ up to 1000 s^−1^ at 298 K. The digitized viscosity data from Fig. 6a in Chandra *et al.*^1^ equal 84 mPa s for a shear rate 1 s^−1^ and 72 mPa s for a shear rate of 1000 s^−1^ at 298 K. The digitized viscosity of [BMIm][BF_4_] from Fig. 3a in Chandra *et al.*^1^ equals 102 mPa s at 298.15 K. Thus, the presented in Fig. 3a viscosity of [BMIm][BF_4_] differs by 21% to 42% from presented in Fig. 6a. The viscosity of [BMIm][BF_4_] reported by Cherecheş *et al.*^4^ is higher than those reported by Chandra *et al.*^1^ by 16% to 35% for shear rate from 1 s^−1^ up to 1000 s^−1^ at 298.15 K. The viscosity of [BMIm][BF_4_] reported by Novak and Britton^20^ is higher than those reported by Chandra *et al.*^1^ by 13% to 32% for shear rate from 1 s^−1^ up to 1000 s^−1^ at 298 K (Fig. 4). Viscosity and density of [EMIm][Gly] obtained by Chandra *et al.*^1^ were compared with literature data in Table 3. The value of viscosity at 298.15 K was obtained by digitalizing Fig. 3b in Chandra *et al.*^1^ Additionally, the range of viscosity of AAILs at 333.15 K reported Chandra *et al.*^1^ was compared with the literature viscosity of [EMIm][Gly] at 333.15 K. The viscosity measured by Chandra *et al.*^1^ was significantly lower than the literature data (Table 3). Moreover Chandra *et al.*^1^ incorrectly cited viscosity of [BMIm][NTf_2_] from,^21^ as 69 mPa s, while in the original paper it was 51.0 mPa s, at 298.15 K. Another incorrect citation is the viscosity of [BMIm][PF_6_] from the work of Qiao *et al.*^22^ Chandra *et al.*^1^ cited a viscosity of [BMIm][PF_6_] equals 450 mPa s at 298.15 K. Whereas, in the original paper, the lowest temperature for which the viscosity is available equals 313.2 K, and the viscosity at this temperature is 109.2 mPa s.^22^ Moreover, Chandra *et al.*^1^ cited viscosity of [EMim][BF_4_], and [EMIm][NTf_2_], but reported values of viscosity were not available in the cited papers.^19,21,22^ In the case of density of [EMIm][Gly], it is difficult to compare its density measured by Chandra *et al.*^1^ with the literature data (see Table 3).

**Table 3:** Viscosity and density of [EMIm][Gly]

Water content (ppm)	*ρ* × 10^−3^ (kg m^−3^)	*η* (mPa s) measurement method	Literature source
***T* = 298.15 K**			
n.a.	1.19^a^	9.7^b^	Chandra *et al.*^1^
		Anton Paar rheometer	
1625	1.1684	272	Bai *et al.*^7^
		Stabinger viscosimetry	
296	1.1547	61.51	Muhammad *et al.*^10^
		Stabinger viscosimetry	
14 400	1.161	171.97	Gouveia *et al.*^9^
		Stabinger viscosimetry	
<100	1.1669	100	Chen *et al.*^8^
0.05 wt% chlorides by Mohr titration		Brookfield vicosimetry	

***T* = 333.15 K**			
n.a.	—	4.3–7.8^c^	Chandra *et al.*^1^
1625	1.1465	44.1	Bai *et al.*^7^
296	1.1327	14.72	Muhammad *et al.*^10^
14 400	1.140	31.24	Gouveia *et al.*^9^
<100	1.1454	16.1	Chen *et al.*^8^
0.05 wt% chlorides by Mohr titration			

a Temperature was not specified.

b Digitalized from Fig. 3b in Chandra *et al.*^1^

c Range of viscosity for [BMIm][Gly], [BMIm][Arg], [EMIm][Gly] and [EMIm][Arg] reported by Chandra *et al.*^1^

#### 3.2.6 Specific isobaric heat capacity

Isobaric heat capacity is an important property in the characterization of heat transfer fluids. While, in the definition provided by Chandra *et al.*^1^ the condition, *i.e.* the constant pressure, was omitted. Chandra *et al.*^1^ discussed values of specific isobaric heat capacity of AAILs, which were related to temperature *ca.* 373.15 K, where the maximum of specific isobaric heat capacity was observed (see Fig. 4c in Chandra *et al.*).^1^ However, if Chandra *et al.*^1^ found the existence of the phase transition due to melting of AAILs at ∼372.15 K, this was related to maximum of the specific isobaric heat capacity. Moreover, in the discussion of results of specific isobaric heat capacity, Chandra *et al.*^1^ did not take into consideration temperature. While, the specific isobaric heat capacity increases with increasing temperature. Literature inspection pointed out that the discussed values of specific isobaric heat capacity of [EMIm][BF_4_] and [BMIm][NTf_2_] are related to 298.15 K. Thus, the comparison of the presented specific isobaric heat capacity of AAILs to those of [EMIm][BF_4_], [BMIm][NTf_2_], as well as of water and ethylene glycol is not justified. However, it should be noted that the specific isobaric heat capacity of AAILs reported by Chandra *et al.*^1^ is greater than that of water at 298.15 K. Additionally, for comparison we used the specific isobaric heat capacity of [BMIm][NTf_2_] at 373.15 K conducted by Hamidova *et al.*^23^ (see Table 4). This shows that if the temperature increase is not accompanied by a phase change, the increase in isobaric heat capacity cannot be as significant as reported by Chandra *et al.*^1^ Besides, Chandra *et al.*^1^ reduced the number of significant digits in the value of the specific isobaric heat capacity reported by Hamidova *et al.*^23^ (see Table 4).

**Table 4:** Specific isobaric heat capacity of ILs selected by Chandra *et al.*^1^

IL	*T* (K)	*c*_p_ × 10^−3^ (J kg^−1^ K^−1^)	Literature source
[EMIm][Arg]	∼373.15^a^	14.15	Chandra *et al.*^1^
[BMIm][Arg]	∼373.15^a^	11.03	Chandra *et al.*^1^
[EMIm][Gly]	∼373.15^a^	7.92	Chandra *et al.*^1^
[BMIm][Gly]	∼373.15^a^	6.30	Chandra *et al.*^1^
[EMIm][BF_4_]	298.15^b^	1.566	Hasen and Abdulmajeed^24^ cited by Chandra *et al.*^1^^d^
[BMIm][NTf_2_]	298.15^c^	1.35	Hamidova *et al.*^23^ cited by Chandra *et al.*^1^
[BMIm][NTf_2_]	298.15	1.35100	Hamidova *et al.*^23^
[BMIm][NTf_2_]	373.15	1.45220	Hamidova *et al.*^23^

a Digitalized from Fig. 4c in Chandra *et al.*^1^

b Taken directly from ref. 24.

c Taken directly from ref. 23.

d It is a ref. 36 instead of ref. 34 in the original paper.

#### 3.2.7 Thermal conductivity

Chandra *et al.*^1^ measured thermal conductivity of ILs and reported results with undefined errors (see Tables S1–S4 in Chandra *et al.*^1^). The authors did not compare their results with the literature data. Taking into account the declared thermal conductivity uncertainty of ±10% for the measurements in the range of 0.2–4 W m^−1^ K^−1^ and ± 0.02 W m^−1^ K^−1^ for the measurements in the range of 0.1–0.2 W m^−1^ K^−1^, the agreement of thermal conductivity of [BMIm][BF_4_] with literature data is excellent (see Table 5). Moreover, it can also be seen that the thermal conductivity of AAILs is higher than those of [BMIm][BF_4_] from 21% for [BMIm][Arg] to 39% for [BMIm][Gly].

**Table 5:** Thermal conductivity of selected ILs

IL	*T* (K)	*λ* (W m^−1^ K^−1^)	Literature source
[EMIm][Arg]	301.04	0.223 ± 0.022	Chandra *et al.*^1^
[BMIm][Arg]	299.27	0.196 ± 0.022	Chandra *et al.*^1^
[EMIm][Gly]	302.32	0.21 ± 0.021	Chandra *et al.*^1^
[BMIm][Gly]	298.29	0.225 ± 0.023	Chandra *et al.*^1^
[BMIm][BF_4_]	301.50	0.162 ± 0.02	Chandra *et al.*^1^
	298.15	0.163 ± 0.01	Nieto de Castro *et al.*^5^
	298.15	0.163 ± 0.01	Ribeiro *et al.*^25^
	297.15	0.177	Minea and Cherecheş^3^

### 3.3. Characterization of ionanofluids

#### 3.3.1 Preparation

Chandra *et al.*^1^ did not provide any specifications of the equipment used in the sonication process, and the parameters of sonication for preparing the INFs were also missing. The authors did not provide the type of ultrasound probe or the name of the manufacturer. There is no information regarding the frequency, amplitude, sonication time, the number of sonication cycles per sample, the energy input per unit mass, or the sonication mode (*e.g.*, continuous or pulsed). It is important to note that high power and prolonged ultrasonication can significantly affect the microstructure of the carbon nanotubes in INFs.^26^ Additionally, samples typically heat up during sonication; however, the authors^1^ did not specify whether their samples experienced heating or whether a cooling bath was used.

#### 3.3.2 Thermogravimetric analysis

**Fig. 5:**
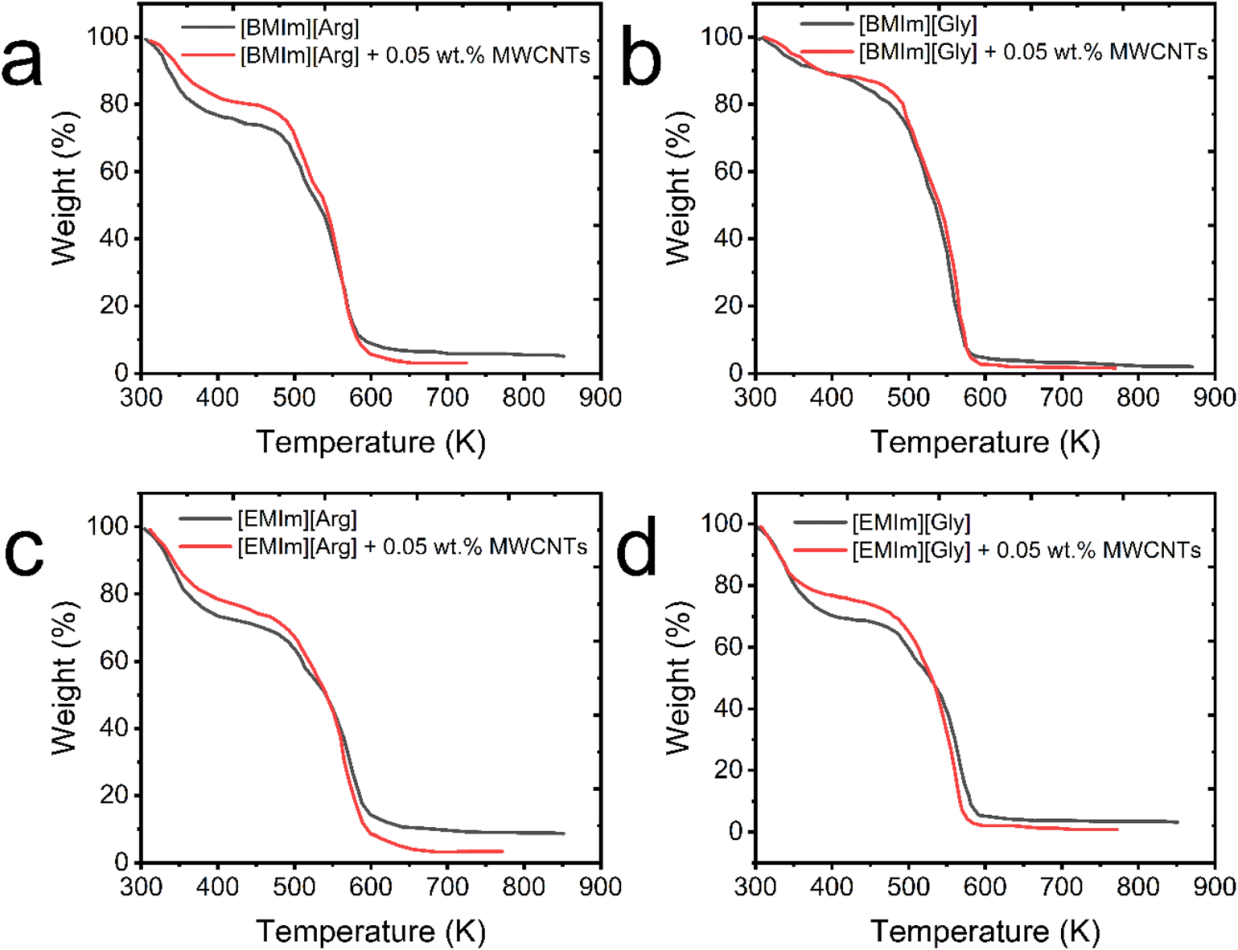
Comparison of TGA curves of AAILs (black curves) and AAIL + 0.05 wt% MWCNTs (red curve) reported by Chandra *et al.*^1^ Publication^1^ is an open access under Creative Commons CC-BY licence.

Based on the TGA profiles of the pure ILs and the INFs, Chandra *et al.*^1^ concluded that MWCNTs enhance the thermal stability of the INFs. This is a fundamentally valid and literature-supported claim.^27–29^ This conclusion was substantiated by the TGA data reported by Chandra *et al.*^1^ (see Fig. 3). The graphs presented in Fig. 5a–d were reconstructed based on Fig. 4a and 8c presented in Chandra *et al.*^1^ using the Digitizer tool available in Origin software. To unequivocally confirm the effect, a comparative plot should have been provided showing both the ionic liquid and its corresponding nanofluid under identical conditions. Instead, the article requires the reader to jump between Fig. 4a and 8c (in Chandra *et al.*^1^) to draw any conclusions. To evaluate the validity of the authors' claim, we superimposed the graphs using graphical software. Even here, difficulties emerged. The analyses were conducted over different temperature ranges, the temperature scale intervals are inconsistent, and the starting temperature of the measurements is not indicated.

These results are in line with published reports. Oster *et al.*^30^ found that the addition of MWCNTs (diameter 13–16 nm, length 1–10 µm, purity >99 wt%) almost linearly increase the onset decomposition temperature in range of 0.5–3.0 wt% of MWCNTs regardless of ILs type, which was also confirmed for other nanoparticle types. On the other hand, both Jóźwiak *et al.*^28^ and Scheller *et al.*^29^ found that the addition of 1.0 wt% of in-house 16 h MWCNTs (16 h – time of synthesis, diameter 60–80 nm, length 780 µm, purity 98 wt%) to 5 different ILs does not significantly alter the first peak temperature derivative, any observed changes were not greater than 2% in decomposition temperature. Similar conclusions were drawn by Hermida-Merino *et al.*,^31^ for graphene nanosheets-based INFs and by Moulefera *et al.*^32^ for graphene oxide-based INFs.

#### 3.3.3 Density

Chandra *et al.*^1^ stated that the density of INFs slightly increased in comparison to base IL. Recently, the effect of MWCNTs loading on density of INFs was discussed by Scheller *et al.*^29^ They observed that the addition of 0.50 wt% in-house 16 h MWCNTs (diameter 60–80 nm, length 780 µm, purity 98 wt%) to [PMpyr][NTf_2_] and [BMpyr][NTf_2_] (1-propyl-1-methylpyrrolidinium bis(trifluoromethylsulfonyl)imide, 1-butyl-1-methylpyrrolidinium bis(trifluoromethylsulfonyl)imide, respectively) resulted in increase in density by 0.20% and 0.23%, respectively, in the temperature range 278.15–363.15 K. A similar observation was made by Jóźwiak *et al.*^28^ The addition of 0.50 wt% of in-house 16 h MWCNTs to 1-ethyl-3-methylimidazolium thiocyanate resulted in an increase in density by 0.19%, at 298.15 K. This effect is further supported by Franca *et al.*^33^ They observed that the addition of 0.50 wt% of MWCNTs (diameter 13–16 nm, length 1–10 µm, purity >99 wt%) resulted in an increase in density of INFs by 0.25%, with no distinction between ILs (among 1-ethyl-3-methylimidazolium dicyanamide, 1-butyl-3-methylimidazolium dicyanamide, 1-butyl-1-methylpyrrolidinium dicyanamide) in the temperature range 293.2–343.2 K. However, the results obtained by Chandra *et al.*^1^ were not in line with the trends noticed in the above-mentioned literature.^28,29,33^ Chandra *et al.*^1^ found that the addition of 0.05 wt% MWCNTs led to an increase of density from 1.14 to 1.19 g cm^−3^ (increase of 4.4%) of [BMIm][Gly]-based INF, and from 1.09 to 1.23 g cm^−3^ (increase of 13%) of [EMIm][Arg]-based INF, assuming that density of IL and INF were measured at the same temperature. This effect is greater by more than one order of magnitude compared to what is reported in the above-mentioned literature.

#### 3.3.4 Viscosity

**Fig. 6:**
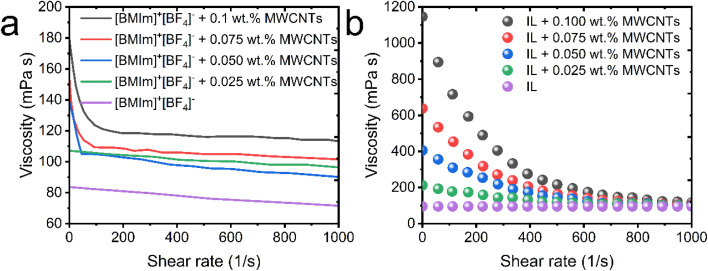
Viscosity variation at different shear rates for [BMIm][BF_4_]-based INFs taken from Chandra *et al.*^1^ (a) and Cherecheş *et al.*^4^ (b), at 298.15 K. Reproduced from ref. 4 with permission from Elsevier, license number 6110141111300. Copyright 2025. Publication^1^ is an open access under Creative Commons CC-BY licence.

The INFs are well known for their non-Newtonian properties. In Fig. 6a, b and 7 in Chandra *et al.*^1^ work, one can clearly see the dependency of the viscosity on the shear rate, albeit Chandra *et al.*^1^ concluded that studied INFs exhibited Newtonian behavior. Moreover, dependencies of viscosity reported by Chandra *et al.*^1^ are unusual, not typically observed in viscosity curves of INFs. Cherecheş *et al.*^4^ studied the similar system of [BMIm][BF_4_] + (0.025–0.01 wt%) MWCNTs (Sigma-Aldrich MWCNTs, purity not stated, dimensions 50–90 nm). They obtained fine viscosity curves at 298.15 K, in parallel plates system with 500 µm measuring gap (compare Fig. 6a with Fig. 6b). Such a dependence is usually observed for INFs systems,^3,11,12,28,29^ with viscosity decreasing non-linearly with increasing shear rate. Moreover, for [EMIm][Gly] + 0.05 wt% MWCNTs a constant viscosity was observed up to 100 s^−1^, the same can be seen for [BMIm][Arg] + 0.05 wt% MWCNTs at a narrower shear rate range (see Fig. 7 in Chandra *et al.*).^1^ This is not typical and it was not commented.

**Fig. 7:**
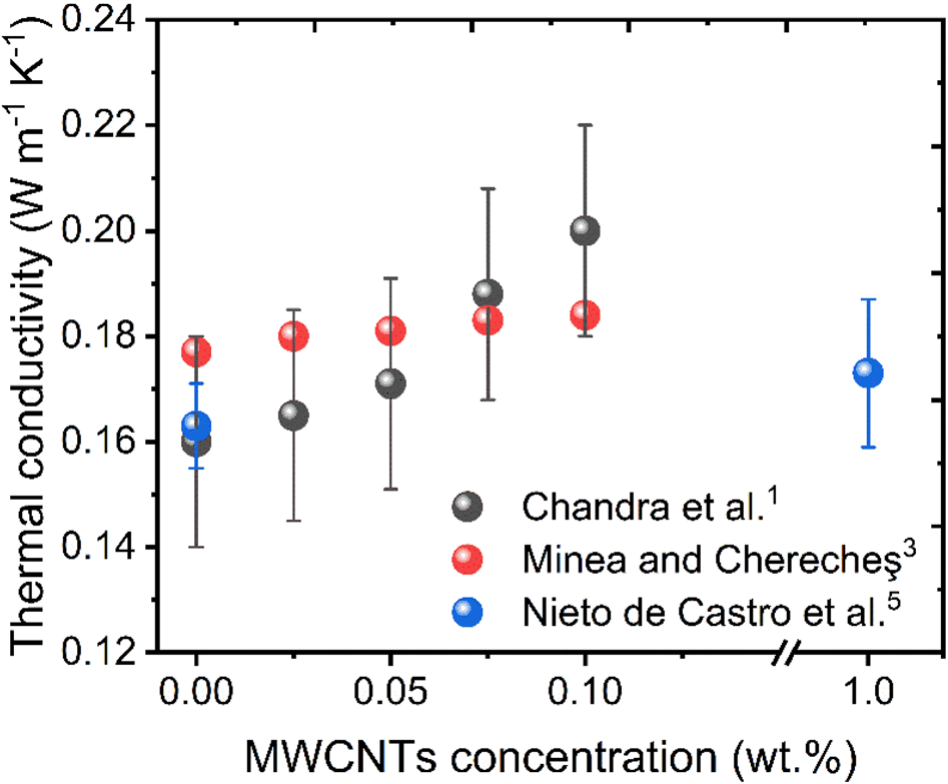
Comparison of thermal conductivity of [BMIm][BF_4_]-based INFs with respect to MWCNTs concentration. In case of Minea and Cherecheş^3^ the error bars are the size of data points and therefore not visible.

Moreover, Chandra *et al.*^1^ approached the modelling of the INFs viscosity with the standard and modified Einstein equation. Unfortunately, the authors did not describe the modification procedure. When applying any model, one should always consider its underlying assumptions. The Eistein model was derived for an ideal Newtonian fluid composed of spherical particles.^34^ Neither of those conditions is fulfilled in the investigated systems, as MWCNTs cannot be treated as spherical particles, and the studied INFs exhibit viscosity variations with shear rate and therefore cannot be regarded as ideal Newtonian fluids.

#### 3.3.5 Differential scanning calorimetry

Chandra *et al.*^1^ investigated thermal behavior of INFs composed of 0.05 wt% MWCNTs and [BMIm][BF_4_] with and without the addition of CTAB (see Fig. 8d and e in Chandra *et al.*).^1^ Both systems exhibited two endothermic thermal events at *ca.* 223 K and 333 K attributed, by the authors, to glass transition and melting. Moreover, the authors stated that they observed an onset of decomposition at 523 K for both [BMIm][BF_4_]-based INFs. Albeit such a conclusion cannot be drawn after inspection of Fig. 8d and e in Chandra *et al.*,^1^ as no such thing is observed. As such, it seems unaffected by the presence of CTAB. Chandra *et al.*^1^ did not investigate pure [BMIm][BF_4_], so the effect of MWCNTs addition cannot be concluded. Shevelvoya *et al.*^35^ investigated thermal behavior of [BMIm][BF_4_] + 12.4 wt% MWCNTs (purity not stated 10–80 nm diameter, length <2 µm) from 80 to 370 K by means of adiabatic calorimeter. The glass transition remained almost the same (182 K) as for the pure IL with no melting observed at higher temperatures (up to 370 K).

**Fig. 8:**
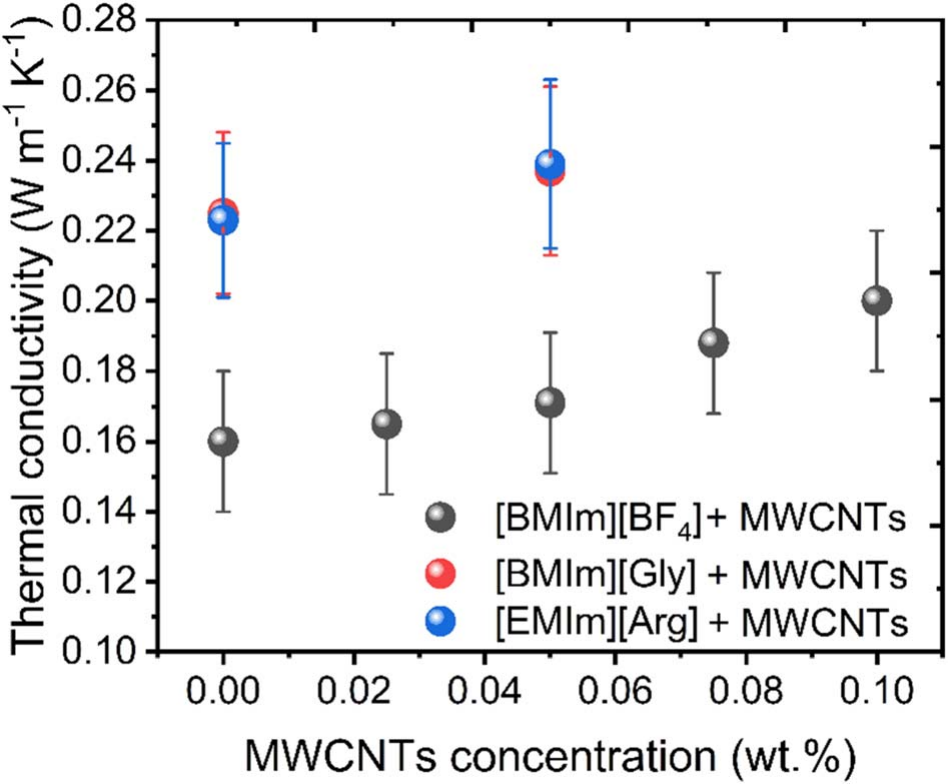
Thermal conductivity of all INFs investigated by Chandra *et al.*^1^ with respect to MWCNTs concentration.

#### 3.3.6 Specific isobaric heat capacity

Chandra *et al.*^1^ reported specific isobaric heat capacity of [BMIm][BF_4_] + 0.05 wt% MWCNTs and [BMIm][BF_4_] + 0.05 wt% MWCNTs + 0.05 wt% CTAB to be 0.4 J g^−1^ K^−1^ and 1.0 J g^−1^ K^−1^, respectively without any consideration to the temperature. Inspection of Fig. 8e in Chandra *et al.*^1^ suggests that reported values of specific isobaric heat capacity related to *ca.* 333 K, which is a maximum in specific isobaric heat capacity connected to thermal event interpreted by Chandra *et al.*^1^ as a melting. Chandra *et al.*^1^ did not report specific isobaric heat capacity of pure [BMIm][BF_4_], rendering the assessment of the effect of the addition of MWCNTs or MWCNTs + CTAB to IL on specific isobaric heat capacity of INFs impossible. The available literature data do not support observed by Chandra *et al.*^1^ specific isobaric heat capacity of [BMIm][BF_4_]-based INFs.^4,35^ Cherecheş *et al.*^4^ studied the effect of the addition of (0.025–0.01 wt%) MWCNTs (purity not stated, dimensions: 50–90 nm) to [BMIm][BF_4_] on the specific isobaric heat capacity of INFs in the temperature range 288.15 to 333.15 K by means of the heat flux DSC (results are reported in Table 6). Shevelvoya *et al.*^35^ investigated specific isobaric heat capacity of pure [BMIm][BF_4_] as well as [BMIm][BF_4_] + 12.4 wt% MWCNTs (purity not stated, 10–80 nm diameter, length <2 µm) from 80 to 370 K using adiabatic calorimeter (results are reported in Table 6).

**Table 6:** Specific isobaric heat capacity of INFs and selected liquids from Chandra *et al.*^1^

IL/INF	*T* (K)	*c*_p_ × 10^−3^ (J kg^−1^ K^−1^)	Literature source
[BMIm][BF_4_] + 0.05 wt% MWCNTs	∼333.15	0.4	Chandra *et al.*^1^
[BMIm][BF_4_] + 0.05 wt% MWCNTs + 0.05 wt% CTAB	∼333.15	1.0	Chandra *et al.*^1^
[BMIm][BF_4_]	333.15	1.752	Cherecheş *et al.*^4^^b^
[BMIm][BF_4_] + 0.05 wt% MWCNTs	333.15	1.779	Cherecheş *et al.*^4^^b^
[BMIm][BF_4_]	334.09	1.696^a^	Shevelvoya *et al.*^35^
[BMIm][BF_4_] + 12.4 wt% MWCNTs	333.09	1.584^a^	Shevelvoya *et al.*^35^
[EMIm][Arg] + 0.05 wt% MWCNTs	∼373.15	10	Chandra *et al.*^1^
Water	Not specified	4.184	Chandra *et al.*^1^
Water	298.15	4.184	Chase^36^
Ethylene glycol	Not specified	2.4	Chandra *et al.*^1^
Ethylene glycol	298.15	2.469	Brzóska *et al.*^37^

a Data from high precision adiabatic calorimeter.

b Digitized from Fig. 11 in Cherecheş *et al.*^4^

Moreover, Chandra *et al.*^1^ reported specific isobaric heat capacity of [EMIm][Arg] + 0.05 wt% MWCNTs to be 10 J g^−1^ K^−1^. This value related to the maximum isobaric heat capacity of this INF at ∼373.15 K (see Fig. 8g in Chandra *et al.*^1^). Such a high value of specific isobaric heat capacity is far greater than any of the known molecular liquids, such as ethylene glycol or even water (see Table 6). Chandra *et al.*^1^ did not comment this phenomenon.

#### 3.3.7 Thermal conductivity

Recently, Minea and Cherecheş^3^ reported the thermal conductivity of INFs composed of [BMIm][BF_4_] and the same MWCNTs loading as in the work of Chandra *et al.*^1^ and in the temperature range of 293.15–333.15 K (see Fig. 7). The dependence of thermal conductivity on MWCNTs concentration differs between the data reported by Chandra *et al.*^1^ and Minea and Cherecheş.^3^ However, these differences are included in uncertaity of thermal conductivity measurements, which are ±10% and *<*1.5% for the results of Chandra *et al.*^1^ and Minea and Cherecheş,^3^ respectively. For comparison, the thermal conductivity of INF composed of [BMIm][BF_4_] and 1 wt% MWCNTs reported by Nieto de Castro *et al.*^5^ was presented in Fig. 7.

Additionally, the available data of thermal conductivity of [BMIm][BF_4_]-based INFs are compared with thermal conductivity of [BMIm][Gly]-based and [EMIm][Arg]-based INFs in Table 7. It is clearly visible, that the addition of 0.05 wt% MWCNTs into [BMIm][Gly] and [EMIm][Arg] resulted in enhancement of thermal conductivity of 5% and 7%, respectively.^1^ This is lower than declared uncertainty of thermal conductivity measurements equals ±10% (see Table 7).

**Table 7:** Thermal conductivity of selected INFs reported by Chandra *et al.*,^1^ Minea and Cherecheş^3^ and Nieto de Castro *et al.*^5^

MWCNTs (wt%)	*T* (K)	*λ* (W m^−1^ K^−1^)	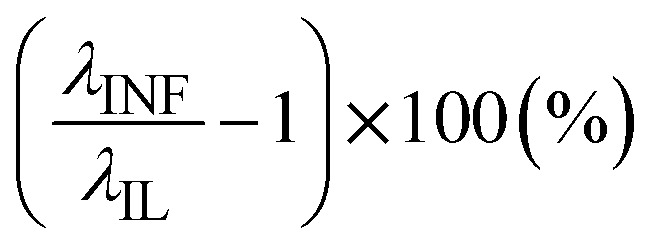	Literature sources
**[BMIm][BF**_**4**_ **]**				
0	301.50	0.162 ± 0.02	—	Chandra *et al.*^1^
	298.15	0.163 ± 0.01	—	Ribeiro *et al.*^25^
	297.15	0.177	—	Minea and Cherecheş^3^
	298.15	0.163 ± 0.01	—	Nieto de Castro *et al.*^5^
0.025	302.10 ± 1.19	0.165 ± 0.02	2 ± 12	Chandra *et al.*^1^
	297.15	0.180^a^	2	Minea and Cherecheş^3^
0.05	301.65 ± 1.19	0.171 ± 0.02	6 ± 12	Chandra *et al.*^1^
	298.15	0.181^a^	2	Minea and Cherecheş^3^
0.075	302.83 ± 1.19	0.188 ± 0.02	16 ± 11	Chandra *et al.*^1^
	297.15	0.183^a^	3	Minea and Cherecheş^3^
0.1	301.63 ± 1.19	0.2 ± 0.02	23 ± 10	Chandra *et al.*^1^
	297.15	0.184^a^	4	Minea and Cherecheş^3^
1		0.173	6	Nieto de Castro *et al.*^5^

**[BMIm][Gly]**				
0	298.29	0.225 ± 0.023	—	Chandra *et al.*^1^
0.05	298.37	0.237 ± 0.024	5 ± 10	Chandra *et al.*^1^

**[EMIm][Arg]**				
0	301.04	0.223 ± 0.022	—	Chandra *et al.*^1^
0.05	298.29	0.239 ± 0.024	7 ± 10	Chandra *et al.*^1^

a Calculated from eqn (1) in Minea and Cherecheş.^3^

Consequently, the comparison of thermal conductivity of AAILs-based INFs with [BMIm][BF_4_]-based INFs is biased, because the thermal conductivity enhancement is affected by thermal conductivity of pure AAILs (see Table 7 and Fig. 8).

## 4. Conclusions

The results presented by Chandra *et al.*^1^ do not support the claims of remarkable thermal conductivity and low viscosity of AAIL-based INFs. Comprehensive studies should include full characterization of the tested substances, both pure components and INFs. A properly selected and well-described methodology for the preparation and characterization of the tested substances, along with descriptions of the equipment, experimental conditions, and measurement uncertainties, should be provided. The results should be critically analyzed and compared with available literature data. The conclusions should be supported by reliable results and, if possible, by literature data and appropriate, adequate theories.

## Conflicts of interest

There are no conflicts of interest to declare.

## Data Availability

No primary research results, software or code have been included and no new data were generated or analysed as part of this comment.
